# Control of a Reassortant Pandemic 2009 H1N1 Influenza Virus Outbreak in an Intensive Swine Breeding Farm: Effect of Vaccination and Enhanced Farm Management Practices

**DOI:** 10.1371/currents.outbreaks.4211b8d6cedd8c870db723455409c0f8

**Published:** 2015-04-13

**Authors:** Lapo Mughini-Gras, Maria Serena Beato, Giorgia Angeloni, Isabella Monne, Filippo Buniolo, Federica Zuliani, Matteo Morini, Alberto Castellan, Lebana Bonfanti, Stefano Marangon

**Affiliations:** Istituto Zooprofilattico Sperimentale delle Venezie (IZSVe), Padua, Italy; National Institute for Public Health and the Environment (RIVM), Centre for Infectious Disease Control (CIb), Bilthoven, The Netherlands; Utrecht University, Faculty of Veterinary Medicine, Department of Infectious Diseases and Immunology, Utrecht, The Netherlands; Istituto Zooprofilattico Sperimentale delle Venezie (IZSVe), Padua, Italy; Istituto Zooprofilattico Sperimentale delle Venezie (IZSVe), Padua, Italy; Istituto Zooprofilattico Sperimentale delle Venezie (IZSVe), Padua, Italy; Istituto Zooprofilattico Sperimentale delle Venezie (IZSVe), Padua, Italy; Istituto Zooprofilattico Sperimentale delle Venezie (IZSVe), Padua, Italy; Istituto Zooprofilattico Sperimentale delle Venezie (IZSVe), Padua, Italy; Freelance DVM, Treviso, Italy; Istituto Zooprofilattico Sperimentale delle Venezie (IZSVe), Padua, Italy; Istituto Zooprofilattico Sperimentale delle Venezie (IZSVe), Padua, Italy

**Keywords:** H1N1, Influenza, outbreak control, reassortant, swine, vaccination

## Abstract

Influenza A viruses in swine cause considerable economic losses and raise concerns about their zoonotic potential. The current paucity of thorough empirical assessments of influenza A virus infection levels in swine herds under different control interventions hinders our understanding of their effectiveness. Between 2012 and 2013, recurrent outbreaks of respiratory disease caused by a reassortant pandemic 2009 H1N1 (H1N1pdm) virus were registered in a swine breeding farm in North-East Italy, providing the opportunity to assess an outbreak response plan based on vaccination and enhanced farm management. All sows/gilts were vaccinated with a H1N1pdm-specific vaccine, biosecurity was enhanced, weaning cycles were lengthened, and cross-fostering of piglets was banned. All tested piglets had maternally-derived antibodies at 30 days of age and were detectable in 5.3% of ~90 day-old piglets. There was a significant reduction in H1N1pdm RT-PCR detections after the intervention. Although our study could not fully determine the extent to which the observed trends in seropositivity or RT-PCR positivity among piglets were due to the intervention or to the natural course of the disease in the herd, we provided suggestive evidence that the applied measures were useful in controlling the outbreak, even without an all-in/all-out system, while keeping farm productivity at full.

## Introduction

Influenza A viruses (IAVs) in swine are among the most commonly isolated pathogens in outbreaks of respiratory disease in domestic pigs worldwide,^1 ^causing considerable economic losses to pig farmers,^2^ and raising concerns about their zoonotic potential.^3^ Continuous circulation of IAVs within swine breeding herds has been documented,^4^ with neonatal pigs playing a crucial role in both virus maintenance in the herd and its spread to other sites.^5^^,^ 6 The predominant IAV subtypes circulating in pigs globally are H1N1, H1N2, and H3N2, though the sporadic identification of other subtypes, including H1N7, H4N6, H9N2, H2N3, H3N3, H5N1, H5N2, and H7N2, has also been reported.^7^^,^ 8 ^,^ 9 ^,^ 10 ^,^ 11 ^,^ 12

Pigs are susceptible to (co-)infection of influenza viruses of swine, avian, and human origin, from which reassortants may appear.^13^ Since the global emergence of the pandemic 2009 H1N1 (H1N1pdm) virus in humans, this has repeatedly been detected in multiple host species, including pigs.^14^^,^ 15 ^,^ 16 ^,^ 17 ^,^ 18 Human-to-swine transmission of H1N1pdm has been documented,^19^ highlighting the evolving nature of influenza viruses and their potential to cross the species barrier. Additionally, transmission experiments have revealed a high susceptibility of pigs to H1N1pdm,^20^ raising concerns about their role in the generation of reassortants between H1N1pdm and the IAVs endemic in swine.

While there are several control strategies for some of the most commonly encountered viral diseases of swine, such as porcine reproductive and respiratory syndrome^21^ and porcine circovirus type 2 infection,^22^ successful interventions for IAVs in swine have not been advocated. Vaccination has been shown to reduce IAV transmission in pigs under experimental conditions,^23^^,^ 24 but the effect of vaccination in defined subpopulations of swine breeding herds remains controversial. For instance, maternally-derived immunity passed from vaccinated sows to their offspring can certainly reduce viral transmission in neonatal pigs,^24^^,^ 25 but this immunity wanes over time,^26^ and modelling studies have predicted that the most common influenza vaccination strategies are ineffective in eliminating influenza virus infection in swine breeding herds.^6^ Besides, there is a paucity of field data and thorough empirical assessments of infection levels in swine herds under different control interventions, resulting in limited understanding of IAV infection dynamics and effectiveness of control measures.

Between August 2012 and August 2013, recurrent outbreaks of respiratory disease were registered in a swine breeding farm in North-East Italy. The virus initially characterized during these outbreaks was a reassortant H1N1pdm containing the neuraminidase (N) and internal genes of the avian-like H1N1.^27^ In August 2012, symptoms of fever and respiratory distress, including anorexia, coughing, sneezing, nasal and ocular discharge, lethargy, and dyspnoea, were observed in 45–60 day-old piglets, with high morbidity (~75%) and mortality (~25%). From August to October 2012, post-mortem examinations of affected pigs revealed signs of interstitial pneumonia with lobular areas of pulmonary consolidation, pleurisy, fibrinous pericarditis, purulent and catarrhal bronchopneumonia. To limit economic losses and to prevent secondary bacterial infections, anti-inflammatory/antipyretic drugs (sodium salicylate and paracetamol) and antibiotics (chosen based on the antibiogram results of bacteria causing secondary infections in the affected animals, namely *Pasteurella multocida*, *Haemophilus parasuis, *and *Streptococcus suis*) were administered. Such interventions had limited or no effect on recovery of respiratory signs. In November 2012, all sows/gilts were vaccinated with Merial’s GRIPOVAC-3, an inactivated vaccine containing H1N1 (Haselünne/IDT2617/2003), H3N2 (Bakum/IDT1769/2003), and H1N2 (Bakum/1832/2000) subtypes, as no specific vaccines for H1N1pdm in swine were registered at that time in Italy. According to manufacturer’s instructions, after primary immunisation with two single-dose injections three weeks apart, a single vaccine booster was administered 14 days prior to farrowing to allow for high colostral immunity. Vaccination led to a perceived reduction of the occurrence of respiratory signs among piglets. However, since March 2013, the occurrence of symptomatic piglets escalated again to the previous levels.

To control the outbreak, an additional vaccination program using a specific vaccine against H1N1pdm was implemented as from October 2013. Indeed, in July 2013, the inactivated vaccine Zoetis’ FluSure-Pandemic, containing the H1N1pdm strain A/California/04/2009 was licensed under derogation in Italy and was then used to vaccinate all the sows/gilts present in the farm. In addition to vaccination, farm management practices were enhanced (see below).

This outbreak provided the opportunity to assess the application of an outbreak response plan based on vaccination and enhanced farm management practices to provide empirical evidence on control strategies for AIVs in swine. The objectives of this study were to describe the control measures implemented since October 2013 in response to this outbreak and to compare virus occurrence in piglets ‒ the subpopulation likely responsible for viral maintenance within the herd^5^^,^ 6 ‒ using a pre-post intervention study design. Additionally, the pattern of maternally-derived immunity in piglets sourced from the affected herd was assessed.

## Methods

Study farm

**The study farm consisted of an intensive, closed-cycle swine breeding farm housing approximately 1,100 sows/gilts and 23,000 piglets located in North-East Italy. This farm supplies ~90 day-old piglets to external fattening farms for the production of traditional Italian ‘Parma’ and ‘San Daniele’ dry-cured hams. Animals are kept under a continuous breeding cycle with production of weekly batches, without applying an all-in/all-out production system. Open and pregnant sows/gilts are housed in individual stalls. Periparturient and lactating sows and neonatal pigs from birth until an average weaning age of 21 days are housed in individual confinement farrowing crates.

Vaccination

**In October 2013, concurrently with GRIPOVAC-3 vaccination, all sows/gilts were vaccinated with FluSure-Pandemic, according to manufacturer’s instructions. For initial immunization, two single-dose injections were administered to all sows and gilts six and three weeks before farrowing and fecundation, respectively. A single vaccine booster was administered three weeks before the first farrow (gilts) or before any subsequent farrow (sows). After four months from the first vaccine administration, all animals received two vaccine shots.

Enhanced farm management

Concurrently with vaccination, farm management was enhanced. Seventy-five additional farrowing crates were installed to allow for the postponement of the weaning age to 28 days in order to decelerate the turnover of piglets entering the ‘wean-to-finish’ barns. In addition, cross-fostering of piglets between sows housed in different farrowing areas was stopped to prevent virus mixing. As for biosecurity, extra cleansing/disinfecting operations at the end of each breeding cycle and mandatory use of disposable coveralls/overshoes for personnel having multiple daily duties in different parts of the farm, were enforced.

Sampling

In October 2013, just before implementing the intervention including vaccination against H1N1pdm, viral circulation was assessed by collecting nasal and saliva swabs from a random sample of 75 symptomatic piglets of 45–60 days of age. Samples were collected in accordance with the European Union Directive 2010/63/EU and processed as described below.

Following H1N1pdm vaccination, maternally-derived antibodies were assessed in piglets born from vaccinated animals. Sera were collected from a convenience sample of two groups of 20 randomly-selected piglets each (hereafter referred to as groups A and B), born one week apart. Six (group A) and five (group B) consecutive sampling events were performed at ~10-day intervals, starting from 30 days of age until ~90 days of age, when piglets were moved out of the farm for fattening elsewhere.

From December 2013 to June 2014, so after implementing the intervention, virus circulation was assessed by collecting nasal and saliva swabs from a random sample of 249 piglets of 35–75 days of age born from vaccinated sows. Seven sampling events were performed. The first five were performed at ~10-day intervals on symptomatic piglets only, whereas the last two were performed two months apart on 50-50% symptomatic-asymptomatic piglets (Table 1).

Serological analyses

**Sera were screened for the presence and titration of antibodies against H1N1, H1N2, H3N2, and H1N1pdm using the haemagglutination inhibition (HI) test as reported elsewhere.^28^ The following antigens were used: A/swine/Italy/267505/2010 (H1N1); A/swine/Italy/284922/2009 (H1N2); A/swine/Italy/312583/2010 (H3N2), and A/Verona-Italy/2810/2009 (H1N1pdm). Samples were pre-treated at 37°C overnight with receptor destroying enzyme (RDE, Sigma Aldrich) and heat-inactivated at 56°C for 30 minutes before use. Sera were tested individually. Four HA units of each virus and a 0.5% chicken red blood cell suspension were used in the test. The initial starting dilution of all sera was 1:10. Sera were considered positive when the antibody titre was ≥1:10.^28^ The geometric mean titre (GMT) was calculated on log_2_ HI titres for each group. Given the focus of this study, only HI data with H1N1pdm antigen are presented. Moreover, as the presence of anti-HA serum antibodies may be associated with protection from clinical disease, piglets were considered to be protected with a titre of ≥1:40 according to available data in the literature.^29^^,^ 30

Virological analyses

Paired specimens of nasal and saliva samples were taken from each piglet. Each nasal swab was placed in vials containing 1 mL of D-MEM medium (Gibco, Life Technologies), while saliva was collected in 2 mL vials after centrifugation at 6,000 g for 30 minutes. Depending on the piglet’s age, health status, pen of origin, and volume of the obtained samples, molecular analyses were performed pooling 2 to 5 samples. When the volume of saliva recovered after centrifugation did not suffice for RNA extraction, the sample was discarded. Viral RNA was extracted from 200 µL of individual or pooled samples using a commercial kit (High Pure RNA Isolation Kit, Roche), according to manufacturer’s instructions. RNA extracts were tested for IAVs using a RT-PCR protocol targeting the matrix (M) gene and an internal control (InType-IC RNA, Qiagen GmbH), which was co-extracted and co-amplified with each sample to account for false negative results due to PCR inhibition.^31^ Subsequently, all IAV-positive samples were analyzed individually by RT-PCR targeting two specific H1N1pdm HA fragments.^31^

Data analysis

Temporal trends in the number of piglets with positive (≥1:10) and protective (≥1:40) maternally-derived antibody titres in each of the two groups of piglets were tested for statistical significance using mixed-effects logistic regression models incorporating a random effect at the individual piglet level to account for non-independence of titres measured in the same piglets over successive sampling events. For each group of piglets, two models were built: one model included the positive (encoded as 1) and negative (encoded as 0) titres as the binary response variable, and the other one included the protective (encoded as 1) and non-protective/negative (encoded as 0) titres as the binary response variable. The sampling event (a variable taking values from 1 to 6 for group A, and from 1 to 5 for group B) was included as a continuous predictor (fixed effect) in both models in order to catch the linear effect of time on titre decay. Higher order polynomials (quadratic and cubic terms) of the sampling event were also tested for, but none was significant so they were not kept in the models. Differences in the proportions of RT-PCR samples positive for H1N1pdm before and after implementation of the control measures were tested for significance using the χ^2 ^test. Statistical analysis was performed using STATA 13 (StataCorp, College Station, USA) and statistical significance was set at p<0.05.

## Results

Maternally-derived immunity

At 30 days of age, all tested piglets showed a positive HI antibody titre for H1N1pdm, whereas 95.0% (95% Confidence Interval [95%CI]: 75.1‒99.9%) and 70% (95%CI: 45.7‒88.1%) of the piglets of group A and group B, respectively, had a protective titre (Figure 1). Titres at 30 days of age ranged from 1:10 to 1:160 (GMT: 49.7) in group A and from 1:10 to 1:640 (GMT: 43.6) in group B (Figure 2).

**Percentages of piglets with a protective (≥1:40) and a positive (≥1:10) maternally-derived antibody titre for H1N1pdm in the two groups of sampled piglets. The first sampling event was performed at 30 days of age and subsequent sampling events were performed at ~10-day intervals. Error bars represent 95% confidence intervals.**
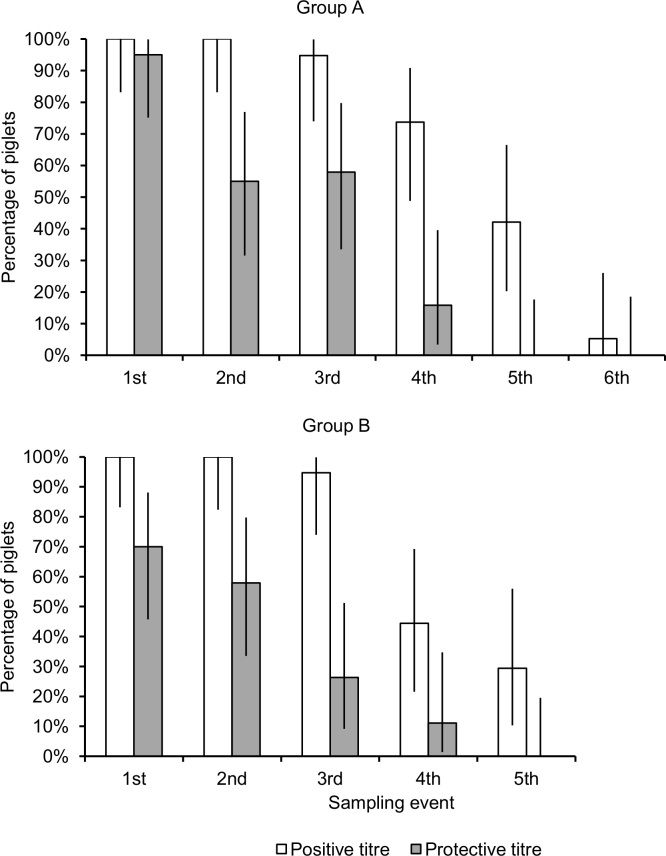

**Geometric mean titres of maternally-derived antibodies in the two groups of piglets. Group A, round dots and segmented exponential trend line; group B, squared dots and solid exponential trend line. The first sampling event was performed at 30 days of age and subsequent sampling events were performed at ~10-day intervals.**
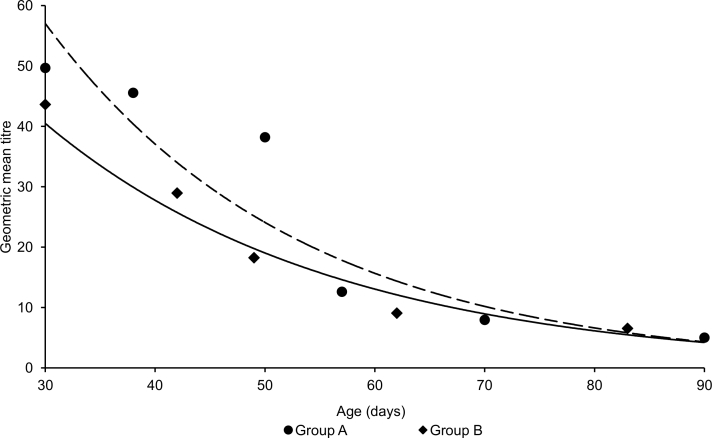

There was a significantly decreasing trend in the proportions of piglets with a positive titre (group A, coefficient = ‒2.38, 95%CI: ‒3.66 to ‒1.12, p<0.0001; group B, coefficient = ‒8.45, 95%CI: ‒15.44 to ‒1.46, p = 0.018) and in those with a protective titre (group A, coefficient = ‒1.97, 95%CI: ‒2.98 to ‒0.96, p<0.0001; group B, coefficient = ‒7.49, 95%CI: ‒12.84 to ‒2.13, p = 0.006) over successive sampling events (Figure 1). The proportion of piglets with a positive titre started decreasing at the third sampling event (50 days of age), when in both groups 94.8% (95%CI: 74.0‒99.9%) of piglets had detectable antibodies. At the fourth sampling event of piglets of group A (57 days of age), 73.7% (95%CI: 48.0‒90.9%) were seropositive to H1N1pdm, and 42.11% at 70 days of age. A similar trend was observed for piglets of group B, though at the fourth sampling event of group B (63 days-of age), 44.0% (95%CI 21.5‒69.2%) of piglets were seropositive. Finally, at 84 and 91 days of age, the last sampling event in either groups, respectively 29.4% (95%CI: 10.3‒56.0%) of piglets in group B and 5.3% (95%CI: 0.1‒26.0%) of piglets in group A still had detectable HI antibodies (Figure 1).

With reference to seroprotection, more than 50% of piglets in both groups showed seroprotective titres. A decrease in the number of piglets of group A with seroprotective titres was observed at 57 days of age, and at 50 days of age for group B. Group A showed a higher number of animals with protective titres than group B, and this was particularly evident at the third sampling event (50 days of age), when group A and B showed 57.9% and 25.0% of seroprotected animals, respectively.

By the end of the post-intervention sampling period, the overall mortality rate in piglets had decreased to 5%, which was the same rate as that normally occurring in the farm before the onset of the outbreak.

Virus detection

Five out of 75 piglets (6.7%, 95%CI: 2.2‒14.9%) sampled before vaccination resulted positive by RT-PCR assays targeting the HA of H1N1pdm. Virus shedding was detected in nasal and saliva swabs in 3 animals and only in saliva for 2 animals. After the intervention, only 1/249 (0.40%, 95%CI: 0.01‒2.21%) piglets tested positive for H1pdm at both nasal and saliva swabs. The difference in the proportion of H1pdm-positive piglets between the pre- and post-intervention periods was statistically significant (χ^2 ^= 11.61, p = 0.003). The positive sample in the post-intervention period was taken at the first sampling performed in December 2013, but since then all other samples tested negative for H1pdm (Table 1).

**Table 1:** Number of positive nasal and saliva swabs out of those tested for the matrix (M) gene of influenza A viruses and for the specific H1pdm gene. All samples were analyzed for the M gene whereas only M gene-positive samples were also tested for H1pdm. *Three piglets were positive at both swab types. **Saliva swabs did not provide enough fluid for analysis. §half of the piglets were asymptomatic. #Samples were analyzed only in pools; 16 pools correspond to 120 individual swabs, 60 of which originated from asymptomatic piglets.

		M gene (positive/total samples)	M gene (positive/total samples)	H1N1pdm HA gene (positive/total samples)	H1N1pdm HA gene (positive/total samples)
Sampling phase	Sampling date	Nasal swabs	Saliva swabs	Nasal swabs	Saliva swabs
Pre-intervention	18/11/2013	10/75 (13.3%)	11/22 (50%)	3/75 (4%)	5/22 (22.7%)
Post-intervention	27/12/2013	6/13 (46.2%)	8/13 (61.5%)	1/13 (7.7%)	1/13 (7.7%)
	8/1/2014	6/20 (30%)	2/18** (11.1%)	0/20 (0%)	0/18 (0%)
	15/01/2014	2/11(18.2%)	0/9** (0%)	0/11 (0%)	0/9 (0%)
	28/01/2014	5/15 (33.3%)	5/12** (41.7%)	0/15 (0%)	0/12 (0%)
	18/02/2014	0/10 (0%)	2/5 (40%)	0/10 (0%)	0/5 (0%)
	1/4/2014	8/60§ (13.3%)	1/44§** (2.3%)	0/60 (0%)	0/44 (0%)
	26/06/2014	5/16# (31.2%)	8/16# (50%)	0/16# (0%)	0/16# (0%)

## Discussion

IAVs are responsible for considerable economic concern for the swine industry and a potential threat to public health. Limited research exists concerning IAV infection levels in swine herds under different control interventions. This study was performed to assess the effect of an outbreak response plan based on vaccination and enhanced farm management practices in an intensive swine breeding farm infected with a reassortant H1N1pdm virus, providing insights into viral circulation and maternally-derived immunity in pig populations under field conditions typical of current commercial farming practices. This is particularly relevant to those pig farms that do not apply an all-in/all-out production system in densely populated swine areas such as northern Italy, where more than 75% of the Italian pig population is located. This study shows that combining direct and indirect control measures, i.e. enhanced biosecurity, postponement of weaning age, ban on cross-fostering of piglets, as well as specific vaccination, may limit (further) virus circulation in intensive swine breeding farms during outbreak situations. H1N1pdm-specific vaccination has probably played a major role in controlling the outbreak, as challenge experiments have showed that inactivated vaccines containing both the HA and N of H1N1pdm do protect pigs from H1N1pdm infection, whereas other IAV vaccines are of limited efficacy.^1^ Yet, virus persistence in the herd was also likely to depend on the type of farm management, as the application of an all-in/all-out production system would have probably sufficed in extinguishing the outbreak, even without vaccination. Since virus persistency in farms would promote the emergence of new variants due to the tendency of influenza viruses for antigenic drift and shift,^14^ the use of specific (and more efficacious) vaccines becomes increasingly important when other control measures based on changes in farming practices are unaffordable or impractical. Vaccination would also decrease the use of drugs for treatment of clinical signs and use of antimicrobials for counteracting secondary infections, and may therefore be considered as an option to protect pigs themselves in addition to prevent antibiotic resistance, to mitigate economic losses, and to reduce exposure to infected pigs, eventually limiting cross-species transfers.

Low levels of H1N1pdm circulation were detected following the implementation of the response plan. The only virological positivity in the post-intervention period occurred right after the implementation of the plan, suggesting that this positivity merely reflects the (last) remainder of the pre-intervention virus circulation levels. This, together with the evidenced elicitation of protective maternally-derived antibody titres, indicated that the implemented measures were effective in lowering the reassortant H1N1pdm virus circulation to undetectabe levels.

As an observational pre-post intervention study on one farm without a control group, it was not entirely possible to determine the extent to which the observed trends in seropositivity or RT-PCR positivity among piglets were due to the intervention or to the natural course of the disease in the herd. This was the main limitation of this study and was due to logistical constraints. However, it is difficult to ignore that the outbreak had been ongoing for more than a year before the situation had suddenly improved following the application of the response plan. Although further investigations are needed to corroborate the effect seen here, our results support, to some extent, the value of H1N1pdm-specific vaccination in sows/gilts in supplementing enhanced farm management practices when dealing with repeated outbreaks of H1N1pdm reassortants in continuous-cycle, intensive swine breeding farms.

As a last point, it has recently been pointed out that SIVs circulating in Italy can infect pig farm workers,^32^ as they showed a significantly higher seroprevalence against swine H1N1 and H1N1pdm viruses than subjects unexposed to pigs. Therefore, since influenza viruses can also spread from humans to pigs,^33^^,^ 34 vaccination against seasonal and pandemic influenza viruses should be enforced in pig farm workers.

## Conclusions

Although our study design was sub-optimal for evaluating the effectiveness of the intervention, we provided suggestive evidence that H1N1pdm-targeted vaccination in sows/gilts, enhanced biosecurity, lengthening of weaning cycles, and constrains in cross-fostering of piglets may prove useful in controlling a reassortant H1N1pdm outbreak in an intensive swine breeding farm without possibility of applying an all-in/all-out system. Maternally-derived immunity was elicited and further virus circulation in piglets dropped to undetectable levels, while keeping farm productivity at full. The present field study also supports the concept that vaccination is part of a wider control strategy and its success may depend on combination of other control measures.

As IAV prevalence is quite high in many swine-rich areas of the world,^5^^,^ 6 ^,^ 4 ^,^ 35 ^,^ 36 ^,^ 37 ^,^ 38 knowledge concerning virus circulation and effect of control measures is needed to have an impact at the herd level. This study provides an empirical assessment of AIV infection levels and colostral immunity in a swine herd under control interventions crucial to reduce both losses for swine producers and the risk to public health.

## Competing Interest

The authors have declared that no competing interests exist.
